# The intercapillary space spectrum as a marker of diabetic retinopathy severity on optical coherence tomography angiography

**DOI:** 10.1038/s41598-022-07128-0

**Published:** 2022-02-23

**Authors:** Noriko Terada, Tomoaki Murakami, Akihito Uji, Kenji Ishihara, Yoko Dodo, Keiichi Nishikawa, Kazuya Morino, Akitaka Tsujikawa

**Affiliations:** Department of Ophthalmology and Visual Sciences, Kyoto University Graduate School of Medicine, 54 Shougoin Kawahara-cho, Sakyo-ku, Kyoto, 606-8507 Japan

**Keywords:** Diseases, Eye diseases, Retinal diseases

## Abstract

Microcirculatory disturbance plays a pivotal role in the pathogenesis in diabetic retinopathy (DR). We retrospectively quantified the total counts and morphological features of *intercapillary spaces*, i.e., intercapillary areas and nonperfusion areas (NPAs), on swept-source optical coherence tomography angiography (SS-OCTA) images and to evaluate their associations with DR severity grades. We acquired 3 × 3 mm OCTA images in 75 eyes of 62 diabetic patients and 22 eyes of 22 nondiabetic subjects. In the en-face superficial images within the central 2 mm, the areas enclosed by retinal vessels were automatically detected. Their total numbers decreased in some eyes with no apparent retinopathy and most eyes with DR, which allowed us to discriminate diabetic subjects from nondiabetic subjects [area under the receiver operating characteristic curve (AUC) = 0.907]. The areas and area/perimeter ratios continuously increased in DR, indicating a continuum between healthy intercapillary areas and NPAs. The number of intercapillary spaces with a high area/perimeter ratio increased according to DR severity, which showed modest performance in discriminating moderate NPDR or higher grades (AUC = 0.868). These quantified parameters of intercapillary spaces can feasibly be used for the early detection of microcirculatory impairment and the diagnosis of referable DR.

## Introduction

Diabetic retinopathy (DR) is a leading cause of vision loss in working people worldwide. Hyperglycemia activates several biochemical pathways and increases the secretion of growth factors and cytokines, which leads to microcirculatory impairment in diabetic retinas^1,2^. In ischemic and hypoxic retinas, neurons are functionally and morphologically perturbed^3,4^, and vascular endothelial growth factor (VEGF) expression results in vascular hyperpermeability and angiogenesis in diabetic macular edema (DME) and proliferative diabetic retinopathy (PDR), respectively^5,6^. This suggests that microcirculatory disturbance plays a pivotal role in the pathogenesis of visual impairment in DR.

Fluorescein angiography (FA) is the gold standard for estimating capillary perfusion by two-dimensional imaging, although optical coherence tomography angiography (OCTA) noninvasively delineates the three-dimensional structure of healthy and diseased chorioretinal vessels^7–9^. The motion contrast signals derived from erythrocyte movements allow us to construct vascular images, whereas classical fluorescent dye represents the flow of plasma^10^. Since erythrocytes deliver oxygen to the retinal parenchyma, the absence of flow signals on OCTA images might indicate hypoxia more directly than capillary nonperfusion on FA images. OCTA can selectively visualize the superficial vascular plexus layer, which allows us to evaluate intercapillary areas throughout the retina, whereas FA delineates these spaces only around the foveal avascular zone (FAZ)^9^. These features of OCTA suggest advantages in the assessment of diabetic microcirculation.

Several pathophysiological mechanisms, e.g., leukostasis, disturbed erythrocyte deformation, and imbalance of the coagulation and fibrinolytic system, lead to transient or permanent disturbance in microcirculation^2,11,12^. The FAZ can be enlarged even in eyes with no apparent retinopathy^13^. The obstruction of retinal capillaries clinically promotes the enlargement of intercapillary areas and the development of nonperfusion areas (NPAs). Since these lesions cannot be discriminated from each other, different researchers have defined NPAs according to different criteria^14–20^.

In this study, we hypothesized the existence of a morphological continuum between intercapillary areas and typical NPAs, both of which are referred to in this study as *intercapillary spaces*, on swept-source (SS-)OCTA images. We investigated how the numbers and morphological parameters of intercapillary spaces are associated with DR severity.

## Results

### Numbers of *intercapillary spaces*

**Figure 1:**
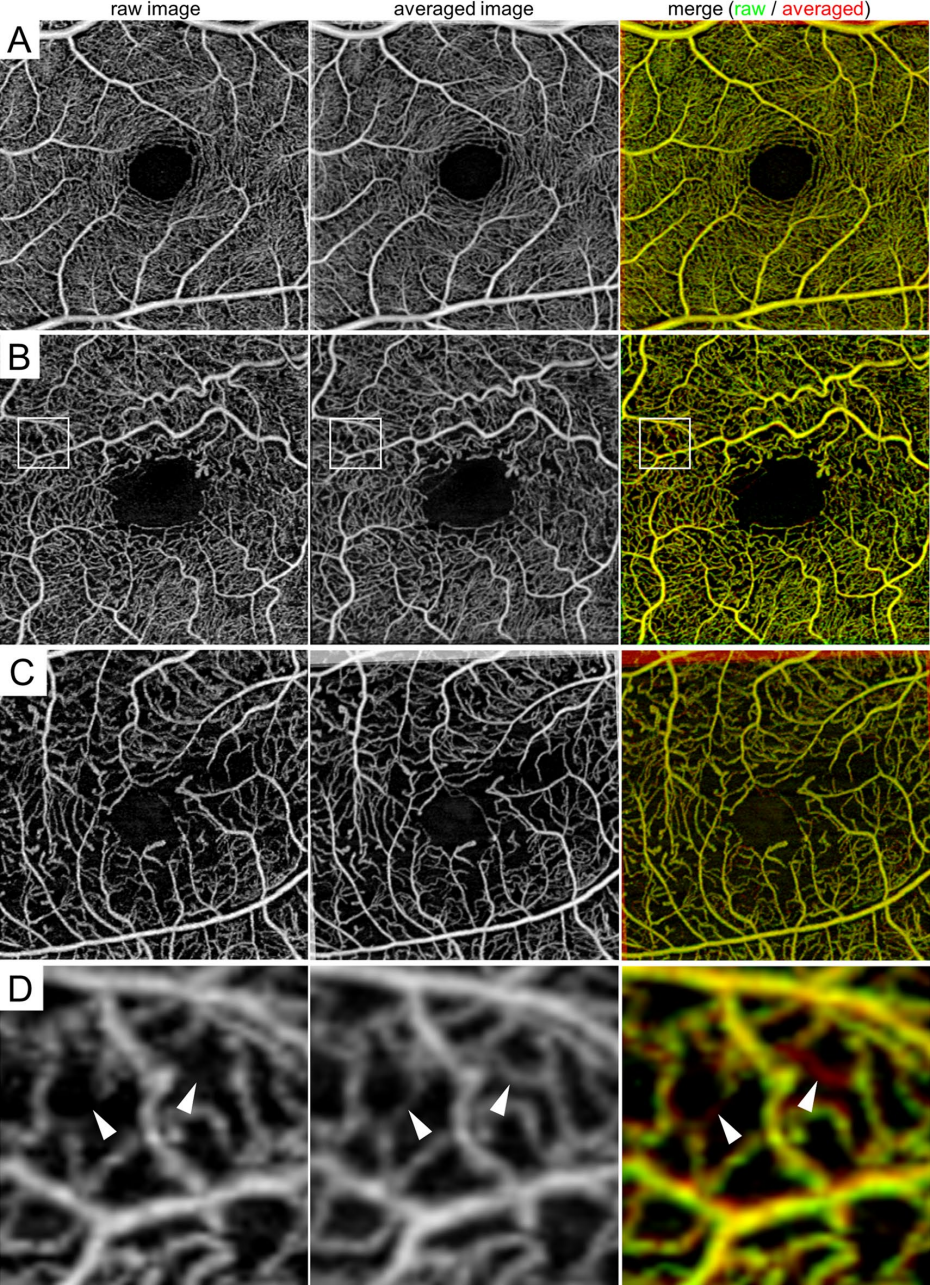
Segmental loss of flow signal and concomitant enlargement of intercapillary spaces on SS-OCTA images in diabetic retinas. (**A**) The flow signals in the raw and averaged images appeared to be similar to each other in the 3 × 3 mm en face image of the superficial layer in a representative nondiabetic subject. (**B**) In contrast, the flow signals are absent segmentally in a representative case of moderate NPDR, resulting in the enlargement of intercapillary spaces. (**C**) The shape of enlarged intercapillary spaces appears to be simple and smooth in a representative case of PDR (**C**). (**D**) Magnified image of the rectangle in panel B. The raw image shows transient loss of flow signals in some retinal capillaries (arrowheads), compared to the uninterrupted vessels in the averaged image. Left column = raw image; middle column = averaged image; right column = merged image.

**Figure 2:**
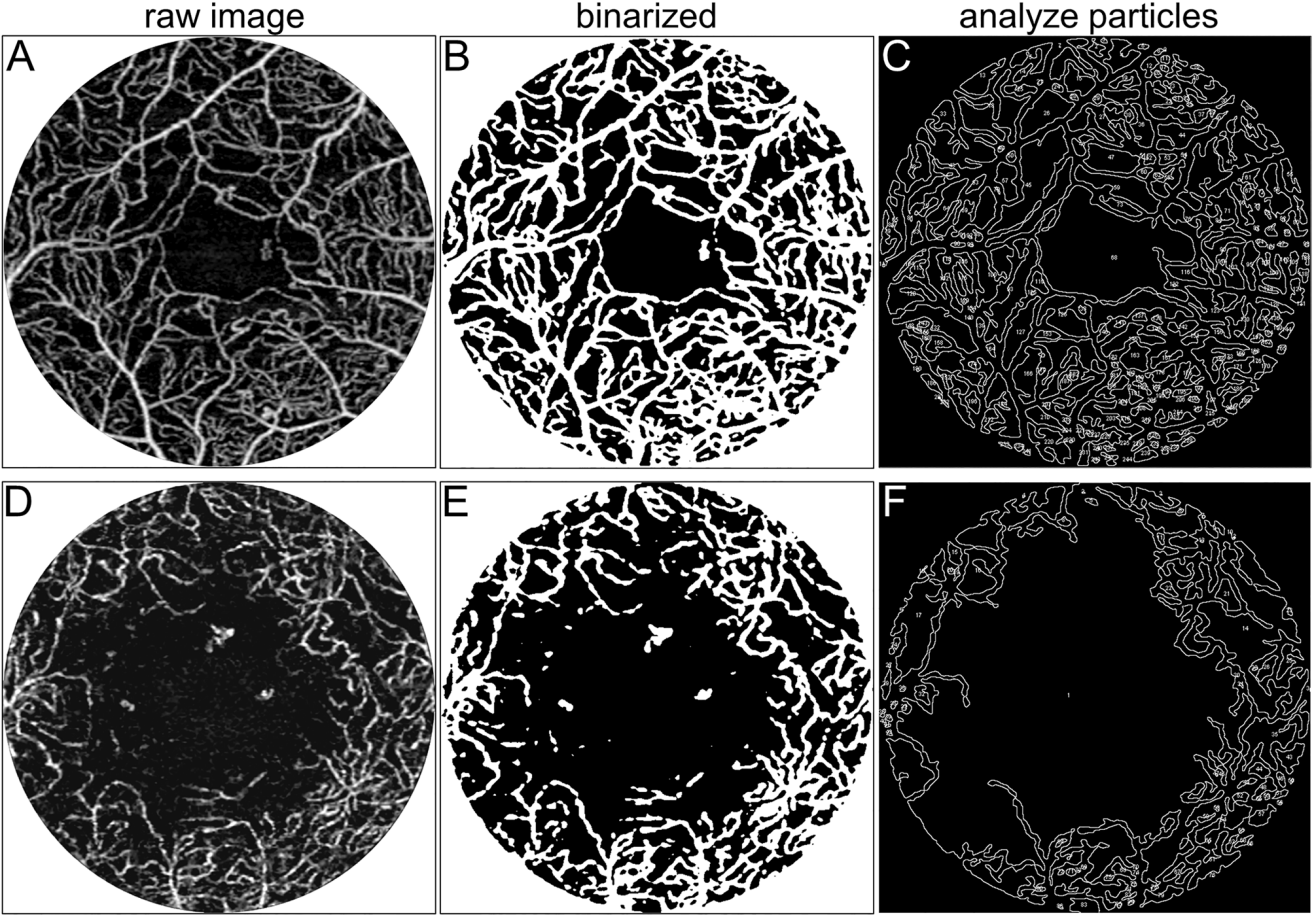
Image processing for the quantification of intercapillary spaces. The binarized image in the central 2 mm circle is applied to the ‘analyze particle’ function in ImageJ, which allows us to measure several parameters of intercapillary spaces. (**A**–**C**) The software can determine and quantify intercapillary spaces in the two-dimensional network of superficial vessels. The counts, mean area, mean perimeter, mean minimum diameter, and mean maximum diameter of the intercapillary spaces are 243, 6.11 × 10^–3^ mm^2^, 0.398 mm, 0.059 mm, and 0.118 mm, respectively. (**D**–**F**) The three-dimensional structure of the deep vascular plexuses does not allow us to detect such spaces definitively.

We evaluated intercapillary spaces in 75 eyes of 62 diabetic patients and 22 eyes of 22 nondiabetic subjects (Fig. 1). The characteristics of the participants are documented in Table 1. The en face OCTA images presented the artery-capillary-vein units in the superficial vascular plexus layer and a nonhierarchical three-dimensional network in the deep layer^21,22^. As a result, the image processing software allowed automatic detection of intercapillary spaces in the superficial layer but not in the deep layer (Fig. 2). Complete obstruction of capillaries resulted in a decrease in their counts and enlarged their areas. In addition, the transient and segmental loss of flow signals, which may occur due to the obstruction of capillaries by erythrocytes, resulted in similar changes (Fig. 1D).

**Table 1:** Characteristics of the patients.

Characteristic	Nondiabetic subjects	No apparent retinopathy	Mild NPDR	Moderate NPDR	Severe NPDR	PDR
Eyes/patients	22/22	14/11	10/7	17/17	16/12	18/15
Age (years)	65(53 to 74)	65(57 to 69)	59.5 (59 to 67)	72(64 to 80)	52(48 to 58)*	54(42 to 58)*
Men/women	10/12	7/4	3/4	14/3	7/5	11/4
Diabetes duration (years)		11 (9 to 15)	10 (7 to 15)	22 (19 to 33)	16.5 (10 to 20)	11 (10 to 20)
Hemoglobin A1c (%)		7.4 (6.7 to 7.7)	7.1 (6.9 to 8.1)	7.2 (7.1 to 7.4)	8.5 (7.0 to 9.0)	8.1 (7.2 to 9.0)
Systemic hypertension (present/absent)	14/8	9/2	3/4	11/6	7/5	10/5
LogMAR VA	− 0.176 (− 0.176 to − 0.079)	− 0.128 (− 0.176 to − 0.020)	− 0.176 (− 0.176 to − 0.079)	− 0.079 (− 0.176 to 0.046)	0 (− 0.079 to 0.155)^†^	0.046 (0 to 0.097)^‡§||^

*P < 0.01 versus moderate NPDR.

^†^*P* < 0.01 versus nondiabetic subjects.

^‡^*P* < 0.001 versus nondiabetic subjects.

^§^*P* < 0.01 versus mild NPDR.

^||^*P* < 0.01 versus moderate NPDR.

**Figure 3:**
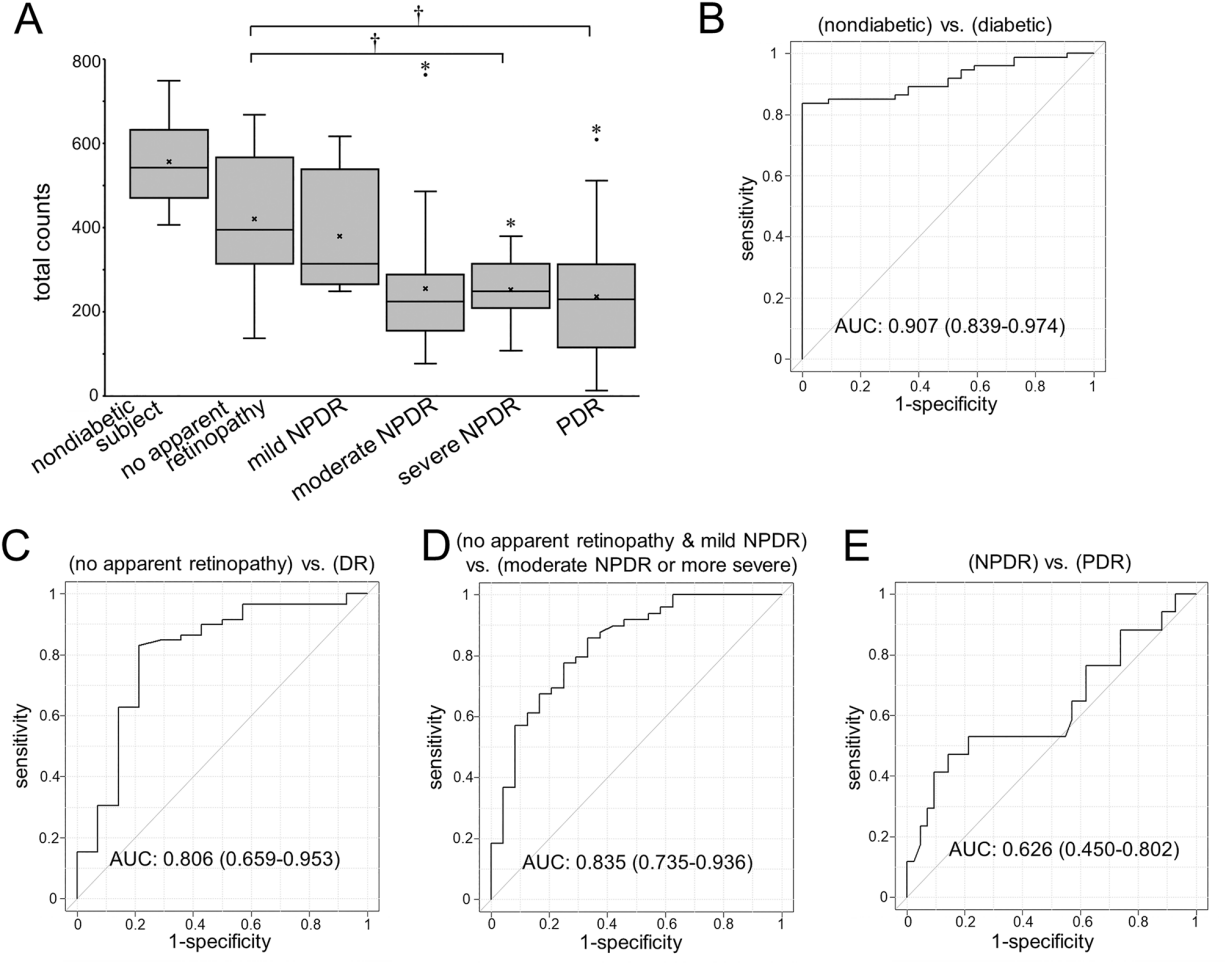
Total numbers of intercapillary spaces in each DR severity grade. (**A**) The total numbers of intercapillary spaces decrease according to DR severity; some eyes with no apparent retinopathy have fewer spaces than nondiabetic eyes. *P < 0.001 versus nondiabetic eyes. †*P* < 0.05 versus no apparent retinopathy. (**B**) The ROC curve reveals the significant power of the total intercapillary space counts to discriminate between the eyes of diabetic and nondiabetic patients. The ROC curves to discriminate DR from no apparent retinopathy (**C**) and referable DR from mild NPDR or no apparent retinopathy (**D**) also show fair power. However, the AUC for the discrimination of PDR from NPDR is not significant (**E**).

We counted the total number of intercapillary spaces and found that the count was significantly decreased in diabetic retinas (542 [473 to 623] vs. 265 [196 to 327] in nondiabetic eyes and diabetic eyes, respectively; *P* < 0.001). Interestingly, some eyes with no apparent retinopathy had almost the same number of intercapillary spaces as the eyes of nondiabetic subjects, and others had fewer spaces (Fig. 3A). No systemic factors were related to the number of intercapillary spaces in eyes with no apparent retinopathy (data not shown). There were fewer intercapillary spaces in eyes with severe nonproliferative diabetic retinopathy (NPDR) or PDR than in eyes with no apparent retinopathy.

### Morphological characteristics of intercapillary spaces

**Figure 4:**
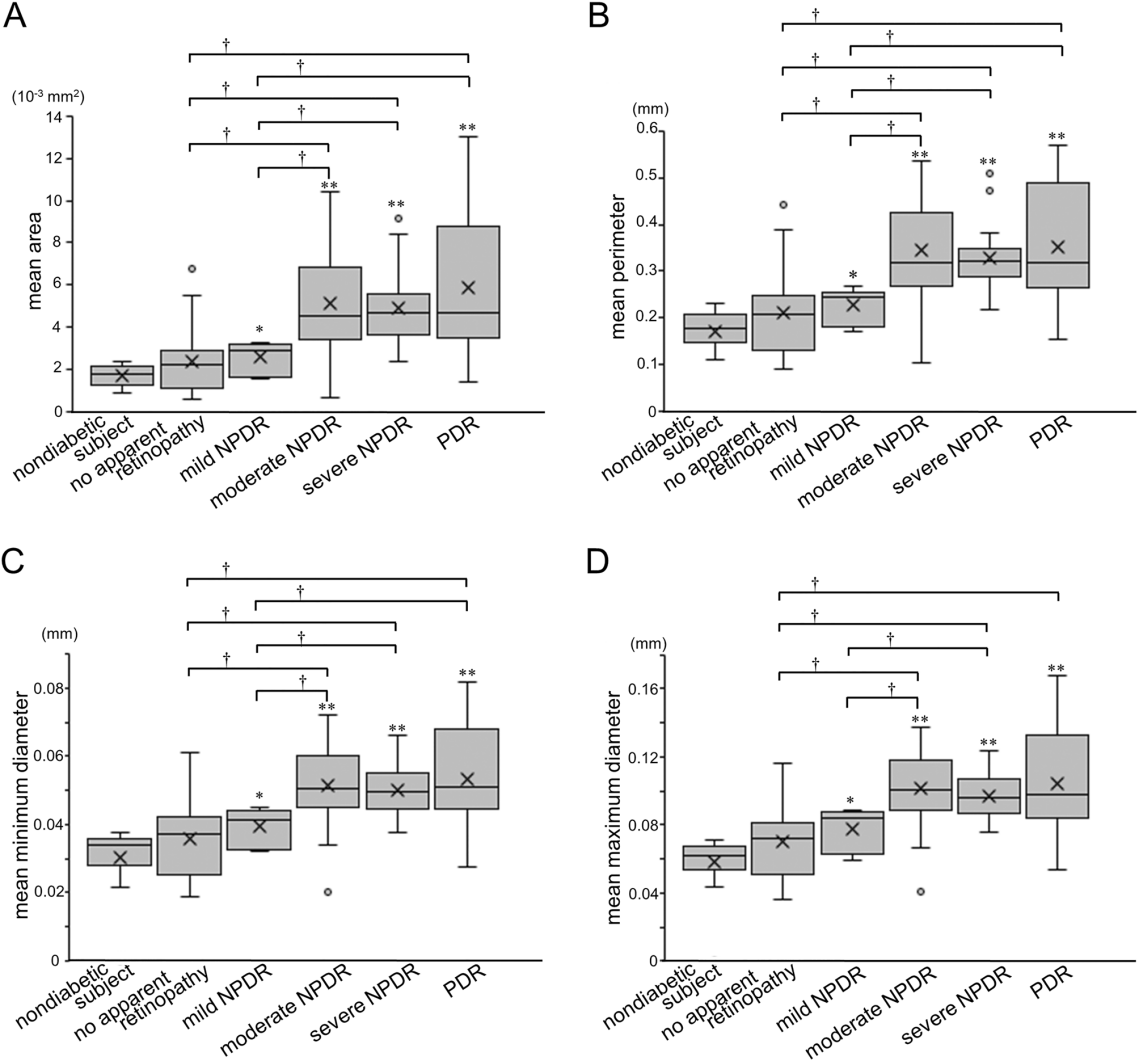
Morphological parameters of intercapillary spaces in each DR severity grade. Mean areas (**A**), perimeters (**B**), minimum diameters (**C**), and maximum diameters (**D**). These parameters are greater in eyes with DR than in nondiabetic subjects. Eyes with referable DR have greater areas and other parameters than those with mild NPDR or no apparent retinopathy. *P < 0.05; **P < 0.01 versus nondiabetic eyes. †*P* < 0.05 versus no apparent retinopathy or mild NPDR.

We quantified four parameters of intercapillary spaces, i.e., the mean areas, perimeters, minimum diameters, and maximum diameters, and found that they were significantly greater in diabetic eyes than in nondiabetic ones (*P* < 0.001 in all comparisons). There were no associations between systemic factors and these parameters (data not shown). In particular, the mean areas of intercapillary spaces were highest in moderate NPDR and higher grades, which is often referred to as referable DR (Fig. 4A). Additionally, the number of intercapillary spaces larger than 0.03 mm^2^ increased (Fig. S1A), and the mean perimeters, minimum diameters, or maximum diameters were also longer in eyes with referable DR than in eyes with no apparent retinopathy or with mild NPDR (Fig. 4B–D).

**Figure 5:**
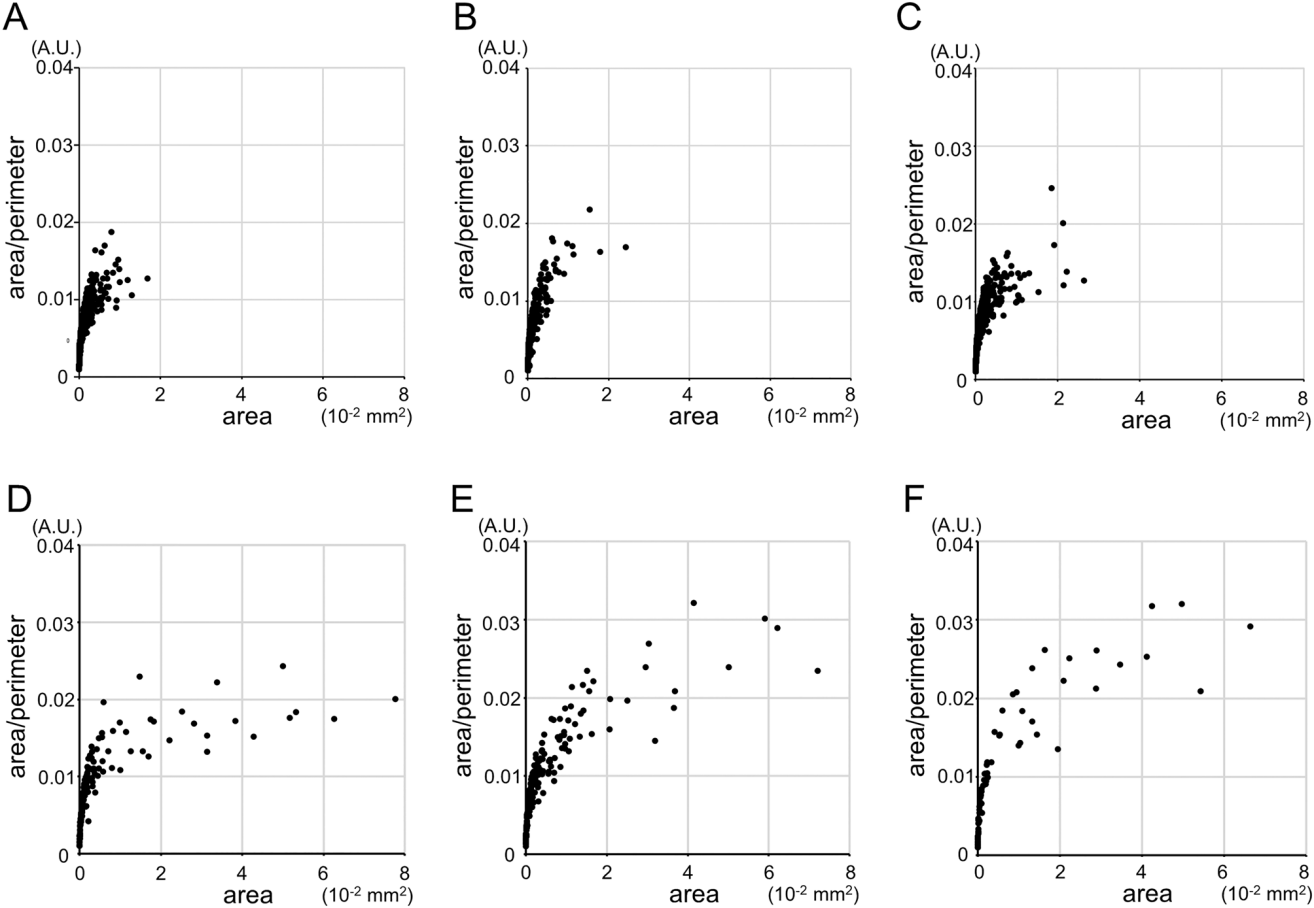
Intercapillary spaces with higher area/perimeter ratios or larger areas in representative cases with referable DR. The intercapillary spaces are small and have low area/perimeter ratios in a representative eye of a nondiabetic subject (**A**), with no apparent retinopathy (**B**), and with mild NPDR (**C**). (**D**) An eye with moderate NPDR had larger intercapillary spaces than the aforementioned groups. (**E**,**F**) An eye with severe NPDR or PDR had some intercapillary spaces with greater area and a higher area/perimeter ratio than those of any other group.

**Figure 6:**
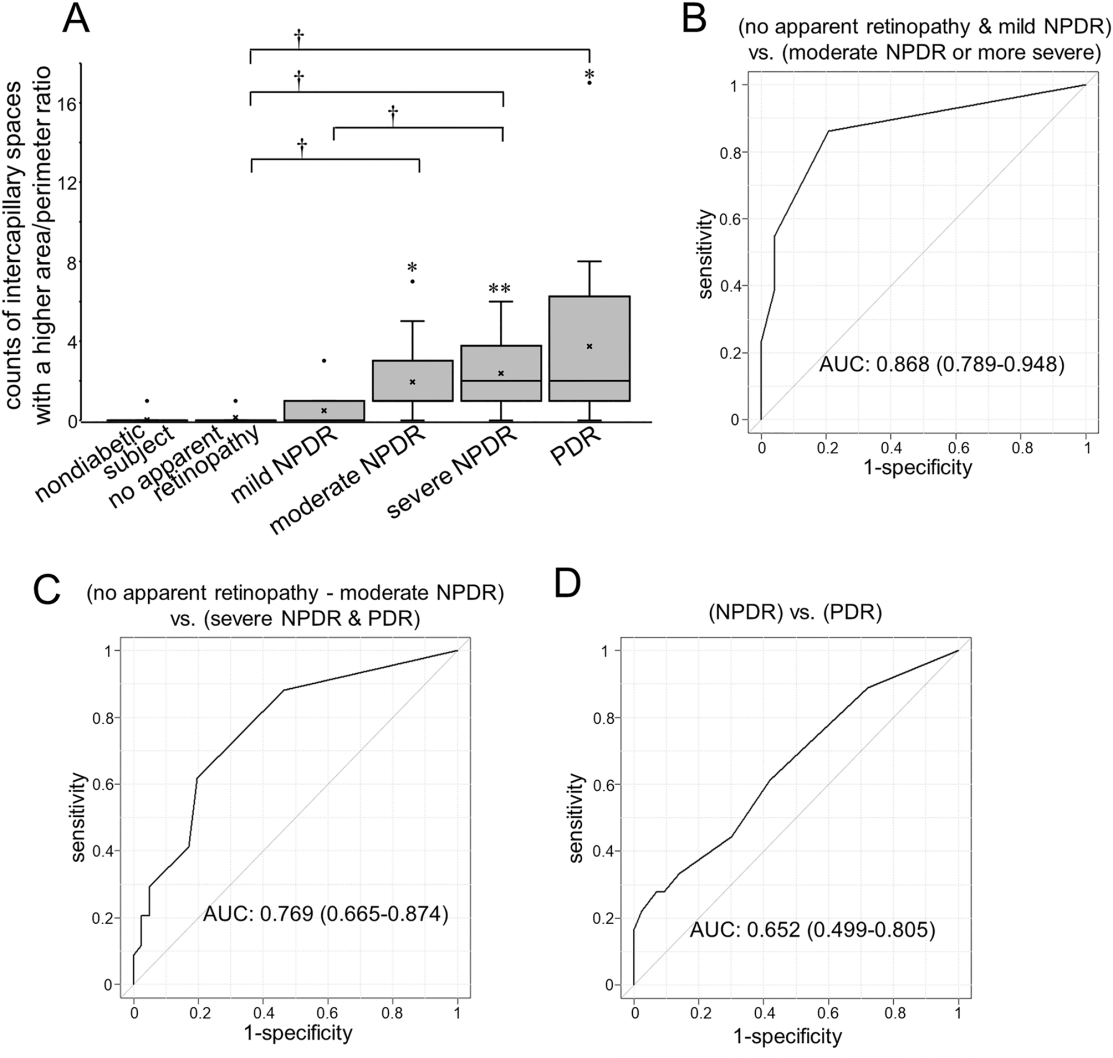
The numbers of intercapillary spaces with an increased area/perimeter ratio. (**A**) The numbers of intercapillary spaces with an area/perimeter ratio greater than 0.025 (A.U.) increase according to the DR severity grades. *P < 0.05, **P < 0.01 versus nondiabetic eyes. ^†^*P* < 0.05 versus no apparent retinopathy or mild NPDR. The ROC curves to discriminate referable DR from no apparent retinopathy and mild NPDR (**B**); severe NPDR and PDR from no apparent retinopathy, mild NPDR, and moderate NPDR (**C**); and PDR from NPDR (**D**). The AUC shows the significant power of intercapillary spaces with an area/perimeter ratio greater than 0.025 (A.U.) to discriminate referable DR from no apparent retinopathy and mild NPDR.

We hypothesized that longer perimeters provide more nutrition and oxygen to neurons in the intercapillary spaces. We therefore calculated the area/perimeter ratio to investigate the effects of vascular remodeling on the nutrient supply in each intercapillary space. Larger intercapillary spaces often had higher area/perimeter ratios in eyes with referable DR (Fig. 5). In particular, the number of intercapillary spaces with area/perimeter ratios greater than 0.025 (A.U.) gradually increased with DR severity (Fig. 6A).

### Clinical relevance of quantitative parameters

We conducted receiver operating characteristic (ROC) curve analyses to investigate the clinical relevance of total counts of intercapillary spaces. The area under the ROC curve (AUC) to discriminate diabetic retinas from nondiabetic retinas was 0.907 (95% confidence interval [CI], 0.839–0.974) (Fig. 3B). The AUC for the discrimination between individual DR grades was smaller (Fig. 3C–E). The mean area and perimeter showed the power to discriminate eyes with referable DR from those with no apparent retinopathy or with mild NPDR (Table 2). In contrast, we did not find the significant power of FAZ parameters (Table S1). We investigated the AUC of the number of intercapillary spaces larger than 0.01, 0.02, 0.03, 0.04, or 0.05 mm^2^, and the number of intercapillary spaces larger than 0.03 mm^2^ had the highest performance in discriminating referable DR (Fig. S1B). In contrast, the AUCs of these morphological parameters were not significant in discriminating PDR from NPDR.

**Table 2:** The AUC of each parameter.

**AUC to discriminate diabetic eyes from nondiabetic ones**	
Mean area	0.865 (95% CI 0.794–0.937)
Mean perimeter	0.861 (95% CI 0.789–0.932)
Mean minimum diameter	0.859 (95% CI 0.787–0.932)
Mean maximum diameter	0.867 (95% CI 0.796–0.937)
**AUC to discriminate eyes with DR from those with no apparent retinopathy**	
Mean area	0.814 (95% CI 0.670–0.958)
Mean perimeter	0.803 (95% CI 0.654–0.953)
Mean minimum diameter	0.796 (95% CI 0.644–0.948)
Mean maximum diameter	0.813 (95% CI 0.666–0.959)
**AUC to discriminate eyes with referable DR from those with mild NPDR or no apparent retinopathy**	
Mean area	0.859 (95% CI 0.764–0.955)
Mean perimeter	0.848 (95% CI 0.749–0.948)
Mean minimum diameter	0.836 (95% CI 0.736–0.936)
Mean maximum diameter	0.838 (95% CI 0.736–0.940)
**AUC to discriminate eyes with NPDR from those with PDR**	
Mean area	0.614 (95% CI 0.446–0.781)
Mean perimeter	0.585 (95% CI 0.415–0.755)
Mean minimum diameter	0.602 (95% CI 0.432–0.772)
Mean maximum diameter	0.596 (95% CI 0.420–0.771)

The number of spaces with an area/perimeter ratio greater than 0.025 (A.U.) had an AUC of 0.868 (95% CI 0.789–0.948) to detect referable DR (Fig. 6B). The ratio had a modest degree of power to discriminate severe NPDR and PDR from moderate NPDR or milder grades (AUC = 0.769 [95% CI 0.665–0.874]; Fig. 6C). Thirty (88.2%) of 34 eyes with severe NPDR or PDR had one or more intercapillary spaces meeting this criterion, while only 19 [46.3%] of 41 eyes with milder grades did (*P* < 0.001). The ratio was not feasible to discriminate PDR from NPDR (Fig. 6D).

## Discussion

In this study, we demonstrated a significant reduction in the total counts and morphological changes of intercapillary spaces on OCTA images in diabetic retinas. Fundoscopic vascular changes were not visible in eyes with no apparent retinopathy, whereas the number of intercapillary spaces on OCTA images was decreased in approximately half of eyes with no apparent retinopathy. This suggests the significance of these counts in the early diagnosis of DR. In addition, other parameters, i.e., the area, minimum diameter, and maximum diameter of intercapillary spaces, were increased in eyes with moderate NPDR or higher grades and showed the power to discriminate referable DR from mild NPDR or no apparent retinopathy. The area/perimeter ratio increased according to DR severity. This suggests that the morphological changes in intercapillary spaces depend, at least in part, on both remodeling and obstruction of retinal vessels. These quantitative parameters showed a continuum rather than definite thresholds between intercapillary areas and typical NPAs in diabetic retinas.

In this study, the areas and perimeters of intercapillary spaces were increased in some eyes with no apparent retinopathy and most eyes with DR. Diabetes increases leukostasis and reduces the deformation of erythrocytes, which may exacerbate the transient plugging of retinal capillaries by erythrocytes^2^. This may explain the segmental loss of flow signals on OCTA images, which leads to the dynamic fusion and splitting of intercapillary spaces in the retinas of early diabetes patients. Frequent fusion causes an increase in the mean area of the spaces. The scatter plots showed that the area/perimeter ratios in most intercapillary spaces were not elevated in eyes with no apparent retinopathy or with mild NPDR. We therefore speculated that the enlargement of intercapillary areas on OCTA images depends on the loss of flow signals in the retinas in the early stages of diabetes.

Both the areas and the area/perimeter ratios increased in referable DR. The ratios may represent morphological simplicity and vascular remodeling. Eyes with moderate NPDR or higher grades often had intercapillary spaces larger than 0.03 mm^2^, which may correspond to pathological NPAs^14–20^. Their continuous distribution on the scatter plots (Fig. 6) suggests a morphological continuum rather than a sharp threshold discriminating between healthy intercapillary areas and pathological NPAs. We therefore propose that the areas encircled by retinal vessels on OCTA images should be considered the *intercapillary space spectrum* for the purpose of quantitative analyses in diabetic retinas. Further studies should be carried out to elucidate the clinical relevance of the intercapillary space spectrum, e.g., its association with visual impairment or DME.

The ROC curve analyses in this study demonstrated that the total number of intercapillary spaces can be a useful early diagnostic sign of DR. The area and area/perimeter ratio are modestly useful diagnostic signs of referable DR. In other words, these parameters of the intercapillary space spectrum may be helpful for diagnosis according to the international DR severity scale, which uses vascular lesions as predictors of PDR development^23^. Enlargement and morphological remodeling of the intercapillary spaces result in oxygen/glucose deprivation in the corresponding neuronal tissues. We speculate that the intercapillary space spectrum could serve as a clinical biomarker of the state of the neurovascular unit in diabetic retinas^24^.

We considered the clinical relevance of intercapillary spaces on OCTA images compared to other modalities or other OCTA-based vascular parameters. Intercapillary areas are clearly delineated around the fovea on FA images, although the vascular structure is not necessarily obvious in the parafovea or perifovea. In addition, fluorescein leakage often reduces the signal-to-noise ratio. In contrast, OCTA can image capillaries in the superficial slab in any retinal area. Among several OCTA parameters, vascular density and FAZ area are often evaluated as indicators of microcirculation^4,13,16,18,25–28^. Small-scale segmental loss of flow signals multiplies the areas of intercapillary spaces and significantly reduces their numbers. This means that the numbers or areas of intercapillary spaces have higher sensitivity than vascular density to detect microcirculation impairment in early diabetes. The FAZ is also a highly sensitive indicator, although its size varies in nondiabetic subjects^29^. The FAZ is a single area, whereas the large number of intercapillary spaces might improve the sensitivity and reproducibility of circulatory disturbance.

This retrospective preliminary study has several limitations. Since many diabetic patients suffer from hypertension, we selected fellow eyes of BRVO as control subjects. Although two groups were balanced in systemic hypertension, this may lead to the selection bias. We excluded eyes with center-involved DME, which may also result in the biased selection. Since we did not exclude large vessels, the enlargement of intercapillary spaces does not necessarily mean the malnutrition. We focused on the intercapillary spaces in the macula; the characteristics of intercapillary spaces elsewhere in the retina remain to be elucidated. The FAZ areas varied on each eye in this study, and might influence the quantified parameters of intercapillary spaces. We employed image processing and statistical software to analyze intercapillary spaces, although further studies could apply deep learning to improve discrimination between individual DR severity grades^30,31^. Additionally, given that this was a single-center study whose subjects were all Asian, prospective multicenter studies should be carried out using other OCTA machines and other image processing algorithms to confirm the reproducibility of the results.

In the current study, we proposed an objective quantification of the *intercapillary space spectrum*, including healthy intercapillary areas and pathological NPAs, on OCTA images of diabetic retinas. Statistical analyses demonstrated the diagnostic significance of our approach and shed light on the microcirculatory pathogenetic processes that affect diabetic retinas in clinical practice.

## Methods

### Patients

We retrospectively reviewed consecutive eyes of diabetic patients for which SS-OCTA images of sufficient quality (signal strength index of 8 or more) were acquired. In addition, the healthy fellow eyes of patients with branch retinal vein occlusion were included as controls in the same period. The exclusion criteria were the presence of center-involved DME; any other chorioretinal disease; other ocular diseases that lead to visual impairment; severe media opacity; previous treatment for macular pathology; photocoagulation within 6 months prior to imaging; cataract surgery within 3 months prior to imaging; any intraocular surgery other than cataract surgery; severe segmentation error in the superficial slab; or axial length < 22 mm or > 26 mm. All research and measurements were performed in compliance with the tenets of the Declaration of Helsinki and with the approval of the Kyoto University Graduate School and Faculty of Medicine Ethics Committee. Written informed consent was obtained from all participants.

### Optical coherence tomography angiography

After measuring the best-corrected decimal visual acuity (VA), we converted it to the logarithm of the minimum angle of resolution (logMAR) VA. After a comprehensive ophthalmic examination, the axial length was measured using partial coherence interferometry (IOL Master, Carl Zeiss Meditec, Inc., Dublin, CA). We acquired three-dimensional optical coherence tomography (OCT) images using Spectralis OCT (Heidelberg Engineering, Heidelberg, Germany), after which we quantified the central subfield thickness (CST). Eyes with CST greater than the thresholds (320 μm or 305 μm for male or female patients, respectively) were diagnosed as center-involved DME^32^.

SS-OCTA images within the nominal 3 × 3 mm square centering on the fovea were obtained using Plex Elite 9000 (Carl Zeiss Meditec, Inc.)^8^. This device operates at 100,000 A-scans/second using a swept-source tunable laser (a center wavelength between 1040 and 1060 nm). After sequential B-scans were acquired at the same position, variations in both intensity and phase information were calculated according to the optical microangiopathy (OMAG) algorithm. The side-by-side arrangement of B-scans yielded three-dimensional OCTA images. The nominal 3 × 3 mm square was obtained with 300 × 300 A-scans, followed by digital conversion to a 1024 × 1024 pixel array for further analysis.

### Intercapillary spaces

Both permanent and transient loss of flow signals enlarged intercapillary areas on OCTA images (Fig. 1). There is not a consensus on the definition of NPAs; different researchers have discriminated NPAs from intercapillary areas using different criteria^14–20^. We therefore hypothesized the existence of a clinical *continuum from intercapillary areas to NPAs* and defined areas enclosed by retinal vessels as *intercapillary spaces* in this study.

We quantitatively evaluated all intercapillary spaces on OCTA images in four steps: (1) the construction of the superficial slab images, (2) the selection of the central 2 mm area, (3) the binarization of retinal vessels, and (4) the automatic quantification of intercapillary spaces. First, we prepared two slab images, i.e., the superficial layer (from the inner limiting membrane [ILM] to the inner plexiform layer [IPL]) and the deep layer (from the IPL to 110 μm above the retinal pigment epithelium [RPE]), according to the default settings of the manufacturer’s software. After the data were exported in TIFF format, the central 2 mm circle was selected using image processing software (Adobe Photoshop, Adobe Systems Inc, San Jose, CA). In some eyes, three images were averaged to detect some retinal vessels with transient loss of flow signals (Fig. 1)^33^. The averaging of OCTA images at different time points delineate some vessels with transient signal loss as well as persistent blood flow. The comparisons between a single and averaged images allow us to confirm transient loss of flow signals in retinal vessels.

We selected a single image but not an averaged image to measure the quantitative parameters of intercapillary spaces, because we planned to evaluate both transient and permanent loss of flow signals. The image was processed to the sequential functions in ImageJ (NIH, Bethesda, MD; Fig. 2). First, Gaussian blur (radius = 2) was applied to reduce speckle noise. Binarized images were created using the Phansalkar adaptive local thresholding method (white pixels represent blood vessels). The black areas circumscribed by vessels in this image were defined as *intercapillary spaces* on OCTA images. After signal inversion (white pixels represent *intercapillary spaces*), the Analyze Particles function was applied to the images to detect each intercapillary space automatically and quantify its area, perimeter, maximum diameter, and minimum diameter (pixels). The pixels were converted to mm or mm^2^. The automatically detected intercapillary space containing the center of the image was defined as the FAZ in this study. After the exclusion of the FAZ, further statistical analyses were applied to the quantified parameters in the intercapillary spaces.

### Statistical analysis

The results are shown as the median (interquartile range [IQR]). The Kruskal–Wallis test with the Bonferroni correction was used for comparisons between groups. Fisher’s exact test or chi-square test was employed to examine the sampling distribution. Spearman’s rank correlatioin coefficient was applied to evaluate the association between two continuous variables. The ROC curve was generated, and the AUC was calculated to evaluate the DR status–discriminating power of the parameters of intercapillary spaces on OCTA images. *P* < 0.05 was considered statistically significant. These statistical analyses were performed using commercial software (PASW Statistics, version 22; SPSS Inc., Chicago, IL).

## Supplementary Information

Supplementary Information.

## Data Availability

The datasets generated and analyzed during the current study are available from the corresponding author on reasonable request.
