# Supplementary material for: The intercapillary space spectrum as a marker of diabetic retinopathy severity on optical coherence tomography angiography
Source: Sci Rep. 2022 Feb 23;12:3089. doi: 10.1038/s41598-022-07128-0 (PMC8866469; doi:10.1038/s41598-022-07128-0)
Supplement: Supplementary file 1 — Supplementary Information. [file 41598_2022_7128_MOESM1_ESM.pdf]

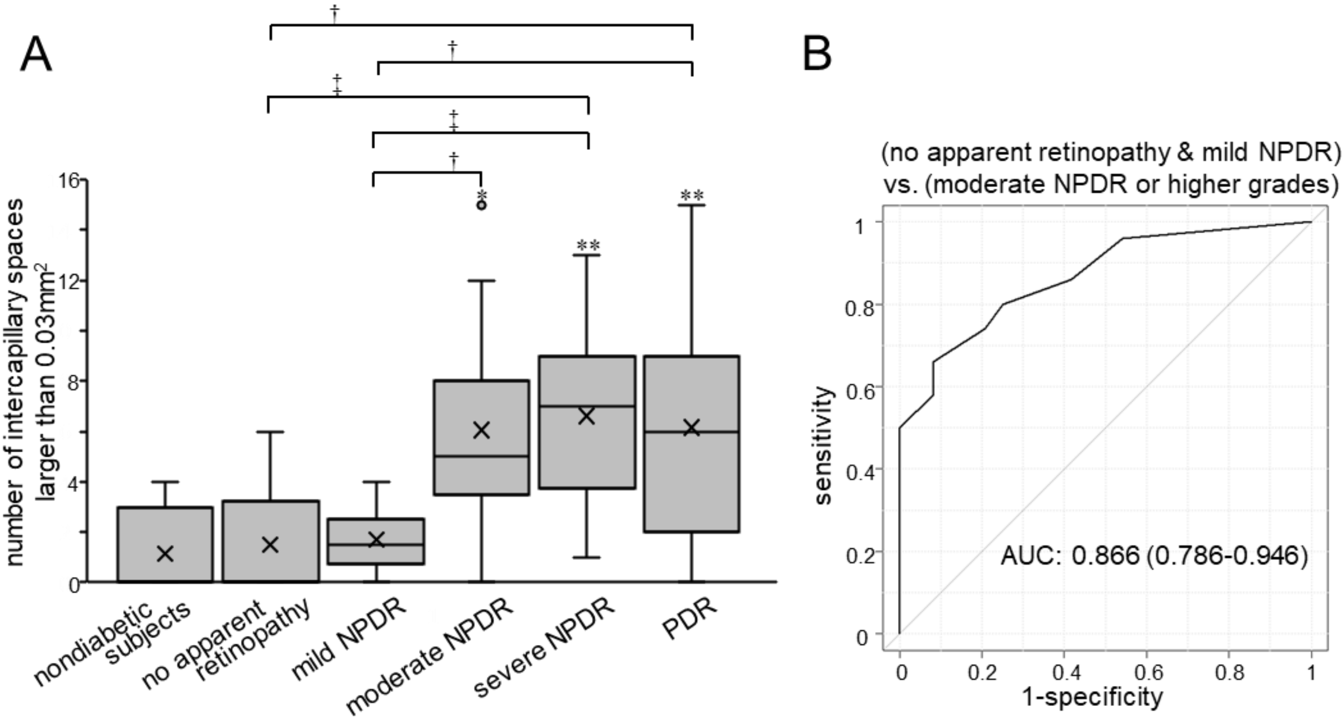

Supplemental Figure 1. Number of intercapillary spaces larger than 0.03 mm^2 in each diabetic retinopathy severity grade.

(A) The numbers of intercapillary spaces larger than 0.03 mm^2 in each diabetic retinopathy (DR) grade. *P<0.01, ** P<0.001 vs. nondiabetic subjects. †P<0.01, ‡P<0.001 vs. no apparent retinopathy or mild NPDR. Eyes with referable DR have more spaces than nondiabetic subjects, eyes with no apparent retinopathy or mild NPDR. (B) The receiver operating characteristic (ROC) curves to discriminate referable DR from no apparent retinopathy and mild nonproliferative diabetic retinopathy (NPDR). PDR = proliferative diabetic retinopathy.

Supplemental Table 1. The AUC of each parameter of the FAZ.

AUC to discriminate diabetic eyes from nondiabetic ones	
area	0.671 (95% CI, 0.564-0.777)
perimeter	0.623 (95% CI, 0.507-0.739)
minimum diameter	0.679 (95% CI, 0.573-0.785)
maximum diameter	0.711 (95% CI, 0.606-0.817)
AUC to discriminate eyes with DR from those with no apparent retinopathy	
area	0.593 (95% CI, 0.444-0.741)
perimeter	0.601 (95% CI, 0.437-0.764)
minimum diameter	0.581 (95% CI, 0.432-0.729)
maximum diameter	0.653 (95% CI, 0.501-0.806)
AUC to discriminate eyes with referable DR from those with mild NPDR or no apparent retinopathy	
area	0.596 (95% CI, 0.462-0.729)
perimeter	0.576 (95% CI, 0.438-0.714)
minimum diameter	0.581 (95% CI, 0.447-0.715)
maximum diameter	0.638 (95% CI, 0.505-0.770)
AUC to discriminate eyes with NPDR from those with PDR	
area	0.514 (95% CI, 0.346-0.683)
perimeter	0.482 (95% CI, 0.314-0.650)
minimum diameter	0.512 (95% CI, 0.342-0.682)
maximum diameter	0.517 (95% CI, 0.346-0.687)
